# Ultra-High-Resolution *in vitro* MRI Study of Age-Related Brain Subcortical Susceptibility Alteration in Rhesus Monkeys at 9.4 T

**DOI:** 10.3389/fnagi.2020.00259

**Published:** 2020-08-14

**Authors:** Qingqing Wen, Hongyi Yang, Jiali Li, Jin Zhang, Haiyang Tong, Qiong Ye, Kai Zhong

**Affiliations:** ^1^High Magnetic Field Laboratory, Hefei Institutes of Physical Science, Chinese Academy of Sciences, Hefei, China; ^2^University of Science and Technology of China, Hefei, China; ^3^Kunming Institute of Zoology, Chinese Academy of Sciences, Kunming, China; ^4^Key Laboratory of Anhui Province for High Field Magnetic Resonance Imaging, Hefei, China; ^5^Center for Excellence in Brain Science and Intelligence Technology, Chinese Academy of Sciences, Shanghai, China

**Keywords:** quantitative susceptibility mapping, iron concentration, brain aging, rhesus monkey, high resolution, 9.4 T

## Abstract

Iron concentration in the brain has been suggested as a biomarker of pathologic neurodegeneration. However, the iron concentration changes in healthy aging as well. This study aimed to quantify the age-related changes in iron concentration in the gray matter of healthy rhesus monkeys using quantitative susceptibility mapping (QSM). Three-dimensional gradient-echo images of 16 female rhesus monkey brains aged between 2 and 26 years were acquired *in vitro*. The susceptibilities in the brain regions of the caudate nucleus (Cd), putamen (Pt), globus pallidus (Gp), and substantia nigra (Sn) were analyzed. The susceptibility varied across different brain regions, with higher levels in the Gp and Sn. Susceptibilities in all analyzed brain regions were linearly correlated with age, yet the plateau period as observed in human brains was absent. This is the first *in vitro* report of the age-related variability of susceptibility in the deep gray matter of rhesus monkey brains at 9.4 T, with an isotropic resolution of 150 μm. Awareness of age-related changes in susceptibility is vital for the establishment of a baseline to facilitate the differentiation of pathologic neurodegeneration from healthy aging in non-human primate studies.

## Introduction

One of the manifestations of brain aging is the disturbance of the physiological balance of iron, which is shown by an increase in the level of non-heme iron in the brain (Hallgren and Sourander, 1958; Daugherty et al., 2015). Iron is a very important trace element in the human body; it participates in oxygen transport, the process of oxygen metabolism, and the synthesis of myelin and neurotransmitters (Piñero and Connor, 2000; Schipper, 2004). The homeostasis of the iron level maintains the normal cell functions of the neural system, yet excessive non-heme iron can increase the vulnerability of cells (Dixon and Stockwell, 2014; Persson et al., 2015). Iron content is strictly regulated in a normal brain. When iron metabolism is disturbed, excessive iron content will induce the generation of nontoxic radicals (Haacke et al., 2010) and lead to oxidative stress injury. With increasing age, iron accumulation in the nervous system increases correspondingly and is preferentially located in the basal ganglia, hippocampus, cerebellar nuclei, and subcortical brain regions (Hallgren and Sourander, 1958; Ghadery et al., 2015). Also, abnormal iron accumulation was observed in some central nervous system diseases, such as Huntington’s disease (HD), Parkinson’s disease (PD), and Alzheimer’s disease (AD; Haacke et al., 2005). Therefore, noninvasive accurate quantification of iron in the brain is crucial for the study of brain aging and neurodegenerative diseases.

In recent years, several noninvasive magnetic resonance imaging (MRI) techniques have been used to study the relationship between age and iron accumulation in human brains, such as field-dependent relaxation rate increase (FDRI; Bartzokis et al., 2004) and susceptibility-weighted imaging (SWI; Liu et al., 2015). Past studies (Bartzokis et al., 1993; Bilgic et al., 2012) showed that FDRI was highly correlated with brain iron levels. However, FDRI obtains R_2_-weighted images under the condition of two different field strengths (e.g., 0.5 T and 1.5 T; Bartzokis et al., 1993; Bilgic et al., 2012), making it time-consuming and susceptible to differences in scanning variability and orientation, thus affecting precision (Persson et al., 2015). Local iron concentration is strongly correlated with magnetic susceptibility values (Bilgic et al., 2012), and SWI uses the magnetic susceptibility information to enhance the contrast between tissues (Haacke et al., 2004; Liu et al., 2015). However, SWI provides susceptibility information qualitatively, but not quantitatively.

To obtain accurate and quantitative susceptibility information, the field map has to be deconvolved, referred to as quantitative susceptibility mapping (QSM; Wang and Liu, 2015). The three steps required to obtain susceptibility maps from phase information are phase unwrapping, background field removal, and deconvolution. Sophisticated harmonic artifact reduction for phase data (SHARP; Schweser et al., 2011) and projection onto dipole fields (PDF; Liu et al., 2011a) are two commonly used methods to remove the background field. There exist many deconvolution methods, such as calculation of susceptibility through multiple orientation sampling (COSMOS; Liu et al., 2009), Bayesian regularization (de Rochefort et al., 2010), and morphology enabled dipole inversion (MEDI; Liu et al., 2012). Several previous studies demonstrated that magnetic susceptibility can provide reliable quantitative measurement of iron content in gray matter (Langkammer et al., 2012; Zheng et al., 2013; Haacke et al., 2015; Sun et al., 2015), with 0.80–1.10 ppb susceptibility per microgram ferritin iron/g wet tissue of human cadaver brain (Zheng et al., 2013) and 0.56–1.30 ppb *in vivo* susceptibility per microgram iron/g wet tissue of human brain (Shmueli et al., 2009; Schweser et al., 2011; Haacke et al., 2015). Also, Langkammer et al. (2012) showed that iron was the dominant source of magnetic susceptibility in subcortical gray matter. Thus, susceptibility was used to estimate the iron content in this study.

In general, higher-spatial-resolution imaging can be obtained at 9.4 T than at 3 T and 7 T. High-resolution MRI can provide detailed anatomical insights into the whole brain, revealing fine structure that cannot be observed in low-resolution images. Also, the high resolution can reduce the partial volume effect, which benefits the segmentation of small regions in the brain, such as the basal ganglia (De Vita et al., 2003). It is well established that the reconstruction of magnetic susceptibility from a field map is an inverse problem. Owing to the ill-posed nature of the inverse problem, streaking artifacts may be present depending on the reconstruction algorithm. High resolution means that more points will fall into the cone of singularity, and noise will be amplified in the process of reconstruction. Therefore, high resolution may increase the artifacts of the susceptibility map. In this study, three different methods, including L_2_-regularization, MEDI, and thresholded K-space division (TKD), were applied to calculate the susceptibility to select the optimal algorithm for the reconstruction of QSM with ultra-high resolution.

Previous studies have shown that iron concentration is relatively higher in the subcortical nuclei (Hallgren and Sourander, 1958; Persson et al., 2015), such as the globus pallidus and striatum (Persson et al., 2015). Both linear and nonlinear relationships between iron concentration estimated by QSM and age have been observed in the human brain (Haacke et al., 2010; Bilgic et al., 2012; Li et al., 2014; Persson et al., 2015). However, QSM studies on non-human primates are rare. Rhesus monkeys and humans share almost 95% of their genes and have similar brain structures, concerning factors such as nuclear organization, projection pathways, and innervation patterns (Magness et al., 2005; Shively and Clarkson, 2009). Owing to their similarity in multiple physiological aspects, rhesus monkeys have been used to study various human diseases, especially neurodegenerative diseases (Camus et al., 2015). This work aimed to find an optimal algorithm for the calculation of an ultra-high-resolution susceptibility map and evaluate the susceptibilities of rhesus monkey brain regions at different ages, to provide a reference for age-related iron deposition in the study of neurodegenerative diseases using rhesus monkeys.

## Materials and Methods

### QSM Theory

Susceptibility reconstruction requires the solution of the equation:

$$F^{-1}DF\chi =\Phi$$

where *F* is the Fourier transform, *D* is the Fourier-domain representation of the unit dipole ($1/3-k_{z}^{2}/|k{|}^{2}$; Schweser et al., 2013), *k* = (*k_x_*, *k_y_*, *k_z_*)^T^ is the coordinate vector in the Fourier domain, $|k{|}^{2}=k_{x}^{2}+k_{y}^{2}+k_{z}^{2}$. *χ* is the unknown susceptibility, and *Φ* is the tissue phase (Bilgic et al., 2014b). L_2_-regularized reconstruction can be regarded as solving Equation (1) *via* the following minimization:

$$\min\left[{\left\|F^{-1}DF\chi -\Phi \right\|}_{2}^{2}+\lambda \cdot {\left\|G\chi \right\|}_{2}^{2}\right]$$

where *G* is the gradient in three dimensions and λ is the regularization parameter. In the algorithm, λ sweeps exponentially from 0.0001 to 1 to determine an optimal value (Bilgic et al., 2013, 2014a).

MEDI introduces a spatial before solving Equation (1):

$$\min\chi \left\|M\nabla \chi \right\|,s.t.{\left\|W(\Phi -F^{-1}DF\chi )\right\|}_{2}\leq \varepsilon$$

where *M* is a binary weighting diagonal matrix derived from the magnitude image (Liu et al., 2013, 2018), *W* is a noise weighting matrix, ∇ represents the gradient operator, and ɛ is equal to the expected noise level. The solution for Equation (3) is:

$$\chi *={\text{argmin}}_{\chi }\left({\left\|M\nabla \chi \right\|}_{2}^{2}+\lambda {\left\|W\left(\Phi -F^{-1}DF\chi \right)\right\|}_{2}^{2}\right)$$

where λ is the regularization parameter specified in the fitting (Liu et al., 2011b).

TKD is a simple and straightforward technique for the reconstruction of susceptibility maps (Shmueli et al., 2009; Wharton et al., 2010; Schweser et al., 2013). Using TKD, the solution of Equation (1) is *χ* = *F*^−1^ *D*^−1^ *FΦ*, where *D*^−1^ is defined as:

$$\begin{array}{l} D{\left(k\right)}^{-1}\\ =\{\begin{array}{cc} {\left(1/3-k_{z}^{2}/|k{|}^{2}\right)}^{-1} & \text{if |1/3}-k_{z}^{2}\text{/|}k{\text{|}}^{2}\text{|}>\delta \\ \mathrm{sgn}\left(1/3-k_{z}^{2}/|k{|}^{2}\right)\cdot \delta^{-1} & \text{otherwise} \end{array} \end{array}$$

Here, sgn represents the signum function and δ is a constant value. The study of Shmueli et al. (2009) has shown that δ in the range of 0.2–0.5 can effectively reduce artifacts.

### Rhesus Monkey Brain Preparation

The animal care and experimental protocols in this study were reviewed and approved by the Ethics Committee of the Kunming Institute of Zoology and the Kunming Primate Research Center, Chinese Academy of Sciences (CAS; AAALAC accredited), and methods were carried out following the approved guidelines. All participating monkeys were raised in the monkey facilities of the Kunming Institute of Zoology, CAS. They were healthy and fed normally without any specific medications or additional iron products. No implants were used for any of these monkeys. They were humanely euthanized owing to suffering from serious accidents. Their main organs were removed and kept following the approved guidelines of the institute. A total of 16 brains of these female rhesus monkeys were included in the study, aged from 2 (born in 2015) to 26 (born in 1991) years. The monkeys were deeply anesthetized with an overdose of sodium pentobarbital (50 mg/kg i.m.) and perfused transcardially with phosphate-buffered saline (PBS) followed by 4% paraformaldehyde (PFA). The brains were removed from the skulls and fixed in a 500-ml solution of 4% PFA. After a fixation period of at least 4 weeks, whole postmortem brains were transferred to a 500-ml solution of PBS for 1 week. All brains were kept and transported at 4°C. Before imaging, each brain was immersed in Fomblin^®^ PFPE (Y LVAC 16/6, Solvay, Italy) within a 3D-printed resin container with maximal size dimensions of 9 cm length, 7 cm width, 8 cm height, and a wall thickness of 2 mm. To minimize unexpected movement during the MRI acquisition, the brain was gently fixed to the container with medical gauze. To remove tiny air bubbles on the brain surface, the sample was placed in a medical vacuum box at 0.03 MPa for 48 h. The brain samples were cleaned with saline solution and then put back into PFA after study.

### Acquisition of MRI Data at 9.4 T

The images of rhesus monkey brains were acquired on a 9.4-T 40-cm MRI system (BioSpec Avance III, Bruker, Karlsruhe, Germany) equipped with a home-built quadrature conformal coil as a transceiver (patent pending). A three-dimensional (3D) gradient-echo sequence was acquired for the analysis of QSM. Ten samples were imaged with the following parameters: repetition time (TR) = 40 ms or 45 ms, flip angle (FA) = 10°, No. of average = 1, matrix = 384 × 384 × 384. A minimum echo time (TE) was used (8.05–14 ms), and the field of view (FOV) was adjusted according to the size of the brain. The scan parameters of the other six samples were: TR = 45 ms, FA = 10°, No. of Average = 1, matrix = 515 × 420 × 354, TE = 13 ms, FOV = 80 mm × 65 mm × 55 mm. The susceptibility maps were reconstructed using both magnitude and phase images.

### QSM Postprocessing and Analysis

**Figure 1:**
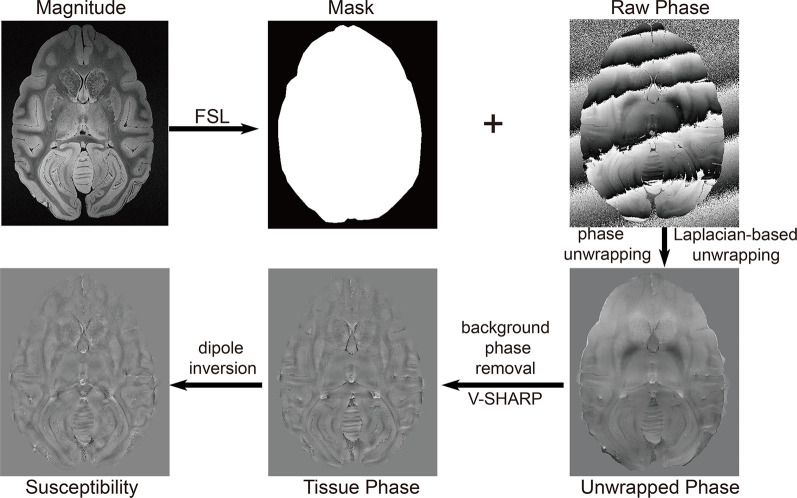
The workflow of the reconstruction of a quantitative susceptibility map using the thresholded K-space division (TKD) algorithm.

Masks were obtained from the magnitude images in FSL (University of Oxford, Oxford, UK). Data postprocessing in this work was performed in MATLAB (MathWorks, Natick, MA, USA) running on a workstation with 32 GB memory and 32 logical processors (Intel^®^ Xeon^®^ CPU E5–2630 v3 @ 2.40 GHz). The phase was unwrapped using a Laplacian-based method (Li et al., 2014). The background phase was subsequently removed using SHARP with the varying spherical kernel (V-SHARP; Kan et al., 2016; Özbay et al., 2017). TKD (Schweser et al., 2013) with thresholds of 0.2, 0.3, and 0.4, MEDI with regularization parameters of 800, 1,000, and 1,500, and the L_2_-regularized algorithm (Bilgic et al., 2014a) were applied to reconstruct the susceptibility maps to achieve the optimal reconstruction algorithm. The postprocessing workflow is illustrated in Figure 1. The quantitative susceptibility maps were calculated from ultra-high-resolution phase images of rhesus monkey brains. The regions of interest (ROIs) in the rhesus monkey brains, including the caudate nucleus (Cd), putamen (Pt), globus pallidus (Gp), and substantia nigra (Sn), were segmented manually on the susceptibility maps. The ROIs of each nucleus were traced on three continuous axial slices in which the border of the structure was well defined. ROIs in bilateral hemispheres were included. Each ROI was drawn five times. The derived average susceptibility from these ROIs was considered as the susceptibility of the corresponding nucleus.

### Statistics

Data are presented as the mean ± standard deviation (STD). The statistics were analyzed in SPSS (IBM Corp., version 16.0, release 2007). The normality of the data was evaluated by the Kolmogorov–Smirnov test. A paired-samples *t*-test was performed between different brain regions. The correlation between susceptibility and age was evaluated using the Pearson correlation coefficient. A linear model of the susceptibility vs. age was obtained by the least-squares fitting. A *p*-value of less than 0.05 was significant.

## Results

**Figure 2:**
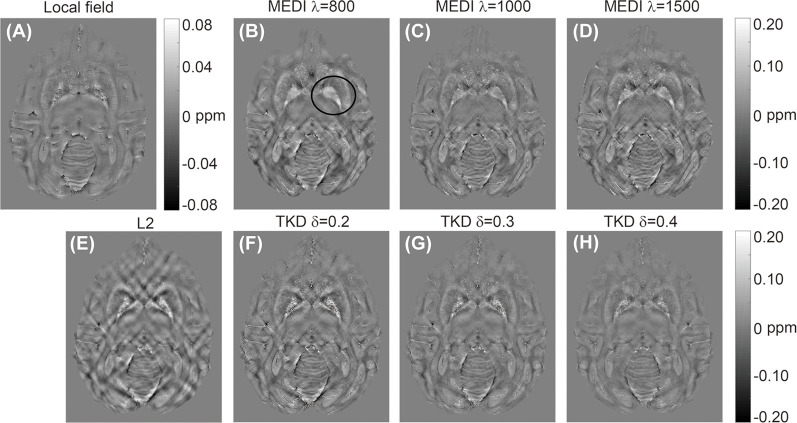
Representative quantitative *in vitro* susceptibility maps of a rhesus brain (19 years old) reconstructed from a local field image at 9.4 T by three different algorithms: **(A)** local field map; **(B–D)** susceptibility maps reconstructed by morphology enabled dipole inversion (MEDI) with regularization parameters of 800, 1,000, and 1,500, respectively; **(E)** susceptibility map reconstructed by L_2_-regularization; **(F–H)** susceptibility map reconstructed by TKD with thresholds of 0.2, 0.3, and 0.4, respectively.

Representative susceptibility maps of the same slice reconstructed using the three different algorithms are shown in Figure 2. The resolution of the image was approximately 0.15 mm × 0.15 mm × 0.15 mm. Obvious streaking artifacts can be observed in the susceptibility maps calculated using MEDI and the L_2_-regularized algorithm. The TKD with thresholds of 0.3 and 0.4 can eliminate the artifact more efficiently than that with a threshold of 0.2. However, the susceptibility reconstructed with TKD decreased with the value of the threshold (Table 1). The MEDI overly smoothed the Gp, in which there were several diamagnetic voxels, resulting in unreliable susceptibility in this region, as shown in Table 1 and in the black circle in Figure 2. Also, the TKD algorithm processed a three-dimensional volume in less than 3 s and the L_2_-regularized algorithm took approximately 10 s to reconstruct the susceptibility map. In comparison, the MEDI algorithm took approximately 5 h to calculate the susceptibility on the same computer. Considering the artifact suppression, reconstruction time, and accuracy of susceptibility, the TKD algorithm with a threshold of 0.25 was selected to reconstruct the susceptibility maps of all rhesus monkey brains *in vitro* in this experiment.

**Table 1:** The averaged susceptibility of caudate nucleus (Cd), putamen (Pt), globus pallidus (Gp), and substantia nigra (Sn) of a 19 years old rhesus brain *in vitro* with different reconstruction algorithms.

Algorithm	Cd (ppm)	Pt (ppm)	Gp (ppm)	Sn (ppm)
MEDI (*λ* = 800)	0.049 ± 0.018	0.035 ± 0.026	0.056 ± 0.035	0.081 ± 0.023
MEDI (*λ* = 1,000)	0.022 ± 0.012	0.017 ± 0.024	0.012 ± 0.015	0.020 ± 0.023
MEDI (*λ* = 1,500)	0.040 ± 0.019	0.023 ± 0.028	0.022 ± 0.025	0.032 ± 0.032
L_2_-regularization	0.016 ± 0.010	0.022 ± 0.021	0.073 ± 0.078	0.088 ± 0.066
TKD (*δ* = 0.2)	0.018 ± 0.008	0.020 ± 0.026	0.073 ± 0.106	0.085 ± 0.088
TKD (*δ* = 0.3)	0.016 ± 0.011	0.018 ± 0.021	0.059 ± 0.083	0.077 ± 0.069
TKD (*δ* = 0.4)	0.011 ± 0.009	0.014 ± 0.017	0.038 ± 0.066	0.060 ± 0.056

*The mean and standard deviation were obtained from a single slice*.

**Figure 3:**
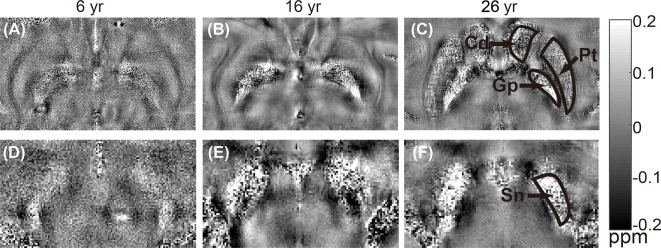
Representative quantitative *in vitro* susceptibility maps of rhesus brains at different ages (6 years, 16 years, and 26 years). Upper row **(A–C)** shows images from the same brain area with regions of interest (ROIs) placed on caudate nucleus (Cd), putamen (Pt), and globus pallidus (Gp). Lower row **(D–F)** shows images from the same brain area with an ROI placed on substantia nigra (Sn).

**Figure 4:**
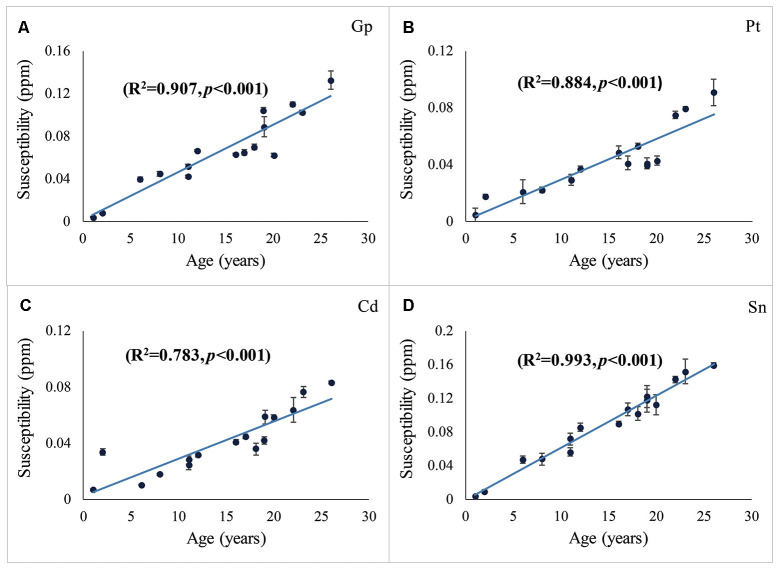
Susceptibilities in the Gp **(A)**, Pt **(B)**, Cd **(C)**, and Sn **(D)** vs. age. The susceptibility vs. age was fitted using the least-squares method. *R*^2^ represents the adjusted *R*-squared. Error bars represent the standard deviation of the mean susceptibility values in the ROIs of the three contiguous axial slices.

The distribution of magnetic susceptibility in the basal ganglia is shown in Figure 3. The susceptibility maps excellently visualize a variety of anatomical structures in the rhesus monkey brains. The susceptibility of different brain regions was different, indicating that the distribution of iron concentration in the brain was in homogeneous. Among the brain regions analyzed, Sn showed the highest iron deposition, followed by Gp, Pt, and Cd (all with *p* < 0.001 in the paired *t*-test except for Cd vs. Pt, for which *p* = 0.647). The average susceptibility values of the four brain regions showed a very strong correlation with age (Gp, *Rho* = 0.938, *p* < 0.001; Cd, *Rho* = 0.890, *p* < 0.001; Pt, *Rho* = 0.908, *p* < 0.001; Sn, *Rho* = 0.987, *p* < 0.001). Figure 4 illustrates the corresponding linear fitting results; as shown in Table 2; the slope (ppm/year) of Sn vs. age (0.00619) was greater than those of Gp vs. age (0.00452), Cd vs. age (0.00300), and Pt vs. age (0.00260), suggesting that the susceptibility of Sn increased more rapidly with age than that of Gp, Pt, or Cd.

**Table 2:** Summary of correlations between susceptibility and age in four analyzed brain regions.

	*χ*(ppm) vs. *age* (years)	*R*^2^	*Rho*	*p*
Gp	*χ* = 0.00452·*age* + 0.00242	0.907	0.938	<0.001
Pt	*χ* = 0.00260·*age* + 0.00246	0.884	0.908	<0.001
Cd	*χ* = 0.00300·*age* + 0.00377	0.783	0.890	<0.001
Sn	*χ* = 0.00619·*age* − 0.00216	0.993	0.987	<0.001

## Discussion

This study compared three different methods of calculating ultra-high-resolution susceptibility maps of rhesus monkey brains *in vitro* and found that TKD was superior to L_2_-regularization and MEDI. Age-related differences in susceptibility were found in the deep gray matter of rhesus monkey brains. This is the first time that iron concentration has been quantitatively estimated in rhesus monkey brains of different ages using QSM at 9.4 T. The differences in iron concentration in different regions of the rhesus monkey brains were significant. We found a linear correlation between age and susceptibility in the basal ganglia of the rhesus monkey brains, which was consistent with the results obtained from atomic absorption spectroscopy in the literature (Hardy et al., 2005).

The quantitative susceptibility maps reconstructed by MEDI and L_2_-regularization display obvious streaking artifacts (Figure 2). In comparison, the TKD algorithm can eliminate the artifacts well. Both MEDI and L_2_-regularization need to be solved by optimal fitting (Liu et al., 2012; Bilgic et al., 2014a), whereas TKD is a threshold processing method based on Fourier space (Schweser et al., 2013), suggesting that the fitting optimization method may not be feasible for the calculation of rhesus monkey brain susceptibility at high resolution. It is noted that there were some diamagnetic voxels in the Gp and Sn of rhesus monkey brains *in vitro*. The MEDI algorithm treated these points as noise and smoothed the image excessively, which resulted in unreliable susceptibility values. With increasing threshold, the artifact suppression of TKD improved, but the susceptibility values decreased. Also, TKD was the fastest among the three algorithms in terms of computing time. Thus, in this experiment TKD with a threshold of 0.25 was adopted.

Although the quantitative correlation between iron content and susceptibility in rhesus monkey brains has not been clarified, many previous works have shown that the susceptibility was positively correlated with iron content in the human brain and have used susceptibility to estimate the relationship between iron concentration and age of humans (Bilgic et al., 2012; Persson et al., 2015). Therefore, in this study, susceptibility was used to estimate the relationship between iron content and age of rhesus monkey brains. The results showed a linear correlation between age and iron deposition in the Cd and Pt of rhesus monkey brains, which is consistent with some previous human brain reports in which iron concentration increased with age (Cherubini et al., 2009; Persson et al., 2015). However, Hallgren and Sourander (1958) found that the iron accumulation in the Cd and Pt of human brains increased slowly until the age of 50–60 years and then entered a plateau period.

It has been found that the susceptibility distribution of the Sn was different across groups of human brains at different ages (Bilgic et al., 2012; Keuken et al., 2017). However, neither Haacke et al. (2010) nor Bilgic et al. (2012) found an age dependency of susceptibility for Sn. In the study by Persson et al. (2015), the susceptibility of Sn rose from the second until the sixth decade of life, followed by a slight decline. This study showed that the iron accumulation in the Sn of rhesus monkey brains was higher than that in the Gp, Cd, or Pt, and the susceptibility in the Sn increased linearly with age. This result, in part, agreed with the result of Hardy’s rhesus monkey experiment (Hardy et al., 2005), but differed partly from results in human brains.

In the deep gray matter of rhesus monkey brains, iron concentration was significantly higher in the Gp than Pt and Cd. Comparing the iron concentrations in Gp of rhesus monkey brains with the iron deposition in Gp of human brains, there is a noteworthy feature. The iron concentration in Gp of rhesus monkey brains increased linearly with age, which was generally consistent with the results of the atomic absorption spectroscopy method (Hardy et al., 2005). In contrast, the relationship between iron deposition in Gp and the age of human brains is a subject of controversy in the literature. Cherubini et al. (2009) reported that the age-dependency of brain iron accumulation in the Gp was linear, whereas Hallgren and Sourander (1958) and Liu et al. (2015) found that the iron concentration in Gp rapidly increased in youth and then approached a plateau. Persson et al. (2015) found a curvilinear age trend, i.e., iron deposition increased rapidly during youth, and then entered a short plateau period followed by a decline after 60 years of age.

Usually, the resolution of 3D human brain images used for QSM calculation was greater than 400 μm or often even 1 mm; for example, 469 μm × 469 μm × 3,000 μm at 1.5 T (Persson et al., 2015), 500 μm × 500 μm × 500 μm at 3 T (Liu et al., 2015), 470 μm × 470 μm × 940 μm and 600 μm × 600 μm × 1,800 μm at 3 T (Schweser et al., 2011), 1,000 μm × 1,000 μm × 2,000 μm at 3 T (Liu et al., 2011a), and 1,000 μm × 1,000 μm × 500 μm at 1.5 T (Liu et al., 2009). The 3D image resolution of the rhesus monkey brains in the study of Hardy et al. (2005) was 1,000 μm × 1,000 μm × 1,000 μm. Most approaches emphasized a high in-plane resolution at the expense of higher section thickness. A lack of high spatial resolution in any dimension can compromise the delineation of small structures. In our study, the spatial resolution of the QSM of rhesus monkey brains was much higher than those in the above-mentioned literature. More details can be observed in the high-resolution images. Moreover, the partial volume effect can influence the accuracy of the ROI susceptibility, as a single voxel at a tissue border may contain two tissue types (Wang and Doddrell, 2001). Additionally, the boundaries of different brain regions in our ultra-high-resolution images were clearer than those in the low-resolution images, improving the accuracy of manual segmentation, which strengthened the reliability of the present study.

There were some diamagnetic voxels in the Gp and Sn of the rhesus monkey brains *in vitro*. We speculate that there are two reasons for these diamagnetic voxels. One might be the permeability of the *in vitro* structure, which led to the entry of other substances into the nuclei. The other might originate from the effect of blood vessels. It was noticed that the susceptibility of Sn in this study was higher than that of Gp, which was not consistent with the previous work (Hardy et al., 2005). We speculated that this inconsistency may be caused by the existence of more diamagnetic voxels in Gp than in Sn, as shown in Figure 3. Future studies are required to systematically investigate the exact reason for the appearance of the diamagnetic voxels.

There are several limitations to this study. First, the sample size is relatively small. In this experiment, although the age range is wide, covering the normal age range of monkeys and corresponding to ages of approximately 6–80 years in humans (Hardy et al., 2005), the quantity of samples is considerably lower than that in most human studies. Second, the ROIs were segmented manually from three represented slices. As the iron concentration may vary within each nucleus, the averaged susceptibility may not represent the susceptibility of the entire nucleus. Future work will focus on a more accurate relationship between iron deposition in monkey brains and age. Third, in addition to the three reconstruction methods used in this study, there have been many other useful reconstruction algorithms used in the human brain QSM. In future studies, a more comprehensive comparison of reconstruction algorithms for high-resolution monkey brains should be performed.

In conclusion, this is the first study to investigate age-related changes in iron concentration of rhesus monkey brains *in vitro* using QSM at 9.4 T. The TKD algorithm can reconstruct ultra-high-resolution QSM better than MEDI and L_2_-regularization algorithms. The iron accumulation in the brains of rhesus monkeys increased linearly with age, yet did not reach a plateau period as observed in human brains. The TKD algorithm was valuable in estimating the iron concentration of normally aged monkey brains, providing a feasible method to further explore the pathologic neurodegeneration caused by disturbed iron accumulation in non-human primates.

## Data Availability Statement

The original contributions presented in the study are included in the article, further inquiries can be directed to the corresponding author/s.

## Ethics Statement

The studies involving nonhuman primate cares and experimental protocols were reviewed and approved by the Ethics Committee of Kunming Institute of Zoology and the Kunming Primate Research Center, Chinese Academy of Sciences (AAALAC accredited), and the methods were carried out in accordance with the approved guidelines.

## Author Contributions

QW is responsible for data analysis and manuscript writing. HY contributed to MRI data acquisition and manuscript editing. JL, JZ, and HT were responsible for animal sample preparation. QY prepared the manuscript. KZ designed and supervised the study. All authors contributed to the article and approved the submitted version.

## Conflict of Interest

The authors declare that the research was conducted in the absence of any commercial or financial relationships that could be construed as a potential conflict of interest.
